# *Durnonovariaodus maiseyi* gen. et sp. nov., a new hybodontiform shark-like chondrichthyan from the Upper Jurassic Kimmeridge Clay Formation of England

**DOI:** 10.7717/peerj.11362

**Published:** 2021-05-11

**Authors:** Sebastian Stumpf, Steve Etches, Charlie J. Underwood, Jürgen Kriwet

**Affiliations:** 1 Department of Palaeontology, University of Vienna, Vienna, Austria; 2 Museum of Jurassic Marine Life, Kimmeridge, Dorset, United Kingdom; 3 School of Earth and Planetary Sciences, Birkbeck College, University of London, London, United Kingdom

**Keywords:** Chondrichthyes, Hybodontiformes, Taxonomy, Kimmeridge Clay Formation, Late Jurassic, Mesozoic, England

## Abstract

A partial skeleton of a hybodontiform shark-like chondrichthyan from the Upper Jurassic Kimmeridge Clay Formation of Dorset, England, is described and designated as a new genus and species, *Durnonovariaodus maiseyi* gen. et **** sp. nov. The holotype and only known specimen, which is represented by disarticulated splanchnocranial elements with associated teeth, a single dorsal fin spine, the pelvic girdle, as well as unidentifiable cartilage fragments, plus countless dermal denticles, exhibits a puzzling combination of dental and skeletal features, providing important new insights into the morphological and ecological diversity of hybodontiforms. *Durnonovariaodus* gen. nov. displays a unique set of dental characters, showing close morphological resemblance to *Secarodus* from the Middle Jurassic of England, which was erected for distinctive, strongly labio-lingually compressed multicuspid cutting teeth originally described as *Hybodus polyprion*. Skeletally, *Durnonovariaodus* gen. nov. resembles *Hybodus* and *Egertonodus* in having a palatoquadrate with a palatobasal process and an ethmoidal articular surface, combined with the possession of dorsal fin spines ornamented with costae. Therefore, and given the absence of any conclusive phylogenetic framework, *Durnonovariaodus maiseyi* gen. et **** sp. nov. is here tentatively referred to Hybodontidae until more complete material becomes available in order to enable a more reliable suprageneric identification. The holotype of *Durnonovariaodus maiseyi* gen. et **** sp. nov. contains two separate pelvic half-girdles, a feature previously considered as evolutionarily primitive among hybodontiforms. However, unfused pelvic half-girdles also occur in the supposedly closely related species *Hybodus hauffianus* and may in fact have been more widely distributed among hybodontiforms than previously thought, thus rendering the phylogenetic utility of separated pelvic half-girdles for inferring hybodontiform interrelationships difficult and unresolved.

## Introduction

Hybodontiformes, which forms a supposed extinct sister group to the elasmobranch crown comprising modern sharks, skates and rays (= Neoselachii sensu Compagno, 1973), represents a speciose clade of Palaeozoic to Mesozoic shark-like chondrichthyans characterized by distinct cranial and dental morphologies, and two dorsal fins supported by heavily ornamented spines exhibiting numerous retrorse denticles arranged along the posterior midline (Maisey, 1978, 1982; Maisey, Naylor & Ward, 2004; Ginter, Hampe & Duffin, 2010; Cappetta, 2012). In addition, a single or double pair of cephalic spines each with a trifid base carrying a prominent hook-shaped spine occurs in males on the skull posterior to the orbit (Maisey, 1982).

First appearing in the Late Devonian, hybodontiforms apparently reached their highest diversity during the Triassic and Jurassic where they flourished and expanded into various ecological niches, ranging from open marine to continental depositional environments (e.g., Rieppel, 1981; Duffin, 1997, 2010; Duffin & Thies, 1997; Rees & Underwood, 2006, 2008; Klug et al., 2010; Fischer et al., 2011; Leuzinger et al., 2015, 2017; Stumpf & Kriwet, 2019). By the Early Cretaceous, hybodontiforms had become almost entirely restricted to marginal marine and brackish water environments before they finally vanished at the end of the Cretaceous (Kriwet & Benton, 2004; Cuny, 2012).

As for elasmobranchs in general, hybodontiforms are characterized by a continuous, life-long tooth replacement resulting in a rich fossil record dominated by isolated teeth, which provide discrete combinations of morphological characters for use in species identification and establishing reliable diagnoses (e.g., Cuny et al., 2008; Cuny, Cavin & Suteethorn, 2009; Rees & Underwood, 2008; Underwood & Cumbaa, 2010; Koot et al., 2013, 2015; Rees et al., 2013; Leuzinger et al., 2017; Szabó & Főzy, 2020). Nevertheless, much uncertainty still surrounds the genus- and higher-level classification of many species, which resulted in the production of a series of different taxonomic and systematic schemes (e.g., Maisey, 1989; Rees, 2008; Cappetta, 2012). This is mainly because our knowledge of the taxonomy and systematics of hybodontiforms is strongly biased towards isolated teeth rather than those found associated with articulated or disarticulated skeletons, which otherwise remain extremely rare and limited to a few localities only, but commonly display important morphological features for inferring phylogenetic interrelationships (e.g., Maisey, 1982, 1983, 1986, 1987, 1989; Maisey, Naylor & Ward, 2004; Lane & Maisey, 2009, 2012; Stumpf et al., 2021). The incomplete nature of the hybodontiform skeletal fossil record consequently precludes deeper insights into their taxonomy and systematics in many cases, and therefore any new information about their skeletal morphology potentially increases our knowledge about their evolutionary history and ecological diversity.

Here, we describe a new hybodontidorm shark-like chondrichthyan, *Durnonovariaodus maiseyi* gen. et sp. nov., from the Upper Jurassic Kimmeridge Clay Formation of southern England based on a partial skeleton with teeth. This new taxon offers important insights into the morphological and taxonomic diversity, as well as the ecology of Mesozoic hybodontiforms and emphasizes the significance of the Jurassic as an important period in the evolutionary history of hybodontiforms before they witnessed a diversity decline and subsequent adaptation to brackish and freshwater environments from the Early Cretaceous onwards.

## Material & Methods

### Geological setting

In England, Late Jurassic shallow epicontinental marine deposits referred to the Kimmeridge Clay Formation crop out at several localities aligned along a narrow, SW–NE trending strip connecting the coasts of Dorset and Yorkshire. In Dorset, the Kimmeridge Clay Formation consists of mudstones and organic-rich laminated shales with intercalated limestones spanning the Kimmeridgian and early Tithonian stages, and was deposited under calm environmental conditions with periods of anoxia (e.g., Hallam, 1987, 1992; Gallois, 2000). These beds are known to have produced a wide variety of fossil vertebrates, including bony and cartilaginous fishes (e.g., Dineley & Metcalf, 1999; Underwood, 2002; Cavin, Forey & Giersch, 2013; Underwood & Claeson, 2019), secondarily marine reptiles (e.g., Benson et al., 2013; Young, Steel & Middelton, 2014; Jacobs & Martill, 2020) and rare remains of pterosaurs and dinosaurs (Martill, Earland & Naish, 2006; Martill & Etches, 2013; O’Sullivan & Martill, 2015).

### Material

**Figure 1:**
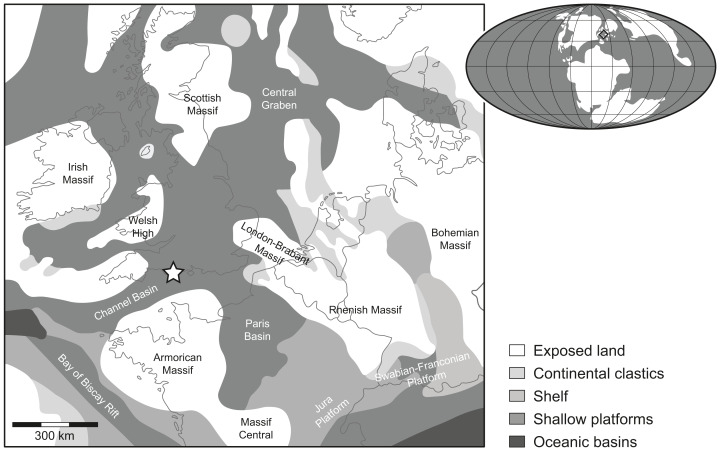
Location map. Rough palaeogeographic reconstruction of the western Tethys during the early Tithonian (modified from Thierry et al., 2000) showing the type locality *Durnonovariaodus maiseyi* gen. et ****sp. nov. near Freshwater Steps, Encombe, Dorset, England (indicated by a star).

The fossil chondrichthyan material described herein consists of an incomplete, disarticulated hybodontiform skeleton preserved on a slab of rock, which was collected by one of us (SE) from early Tithonian beds referred to the *Pectinatites pectinatus* ammonite zone accessible near Freshwater Steps, Encombe, Dorset (Fig. 1). The specimen is housed and curated in the Museum of Jurassic Marine Life (MJML) of Kimmeridge in Dorset, which was built to house the lifetime collection of one of us (SE) (see Noé, Gómez-Pérez & Nicholls, 2019), and was briefly described and referred to *Planohybodus peterboroughensis* Rees & Underwood, 2008 by Underwood (2020), together with additional specimens housed in the MJML, whose detailed descriptions will shed further light on the diversity of Kimmeridge Clay Formation hybodontiforms.

### Methods

Photographs presented in the text were obtained by digital macro- and micro-photography using a Nikon D5300 DSLR camera with either an AF-S DX NIKKOR 18–140 mm f/3.5–5.6 G ED VR or an AF-S DX Micro NIKKOR 40 mm f/2.8G lens. All photographs were rendered utilizing the software package Adobe Photoshop CC 2018 and the accompanying figures were created using Adobe Illustrator CC 2018.

Descriptive terminologies used for the skeletal morphology correspond to those of Maisey (1982), and terminologies for the dental morphology largely follow that of Cappetta (2012).

### Nomenclatural acts

The electronic version of this article in Portable Document Format (PDF) will represent a published work according to the International Commission on Zoological Nomenclature (ICZN), and hence the new names contained in the electronic version are effectively published under that Code from the electronic edition alone. This published work and the nomenclatural acts it contains have been registered in ZooBank, the online registration system for the ICZN. The ZooBank LSIDs (Life Science Identifiers) can be resolved and the associated information viewed through any standard web browser by appending the LSID to the prefix http://zoobank.org/. The LSID for this publication is: urn:lsid:zoobank.org:pub:21199B53-9E93-44FF-987C-EDC115E8AA88. The online version of this work is archived and available from the following digital repositories: PeerJ, PubMed Central and CLOCKSS.

## Results

### Systematic palaeontology

Class CHONDRICHTHYES Huxley, 1880

Subclass ELASMOBRANCHII Bonaparte, 1838

Order HYBODONTIFORMES Maisey, 1975

Family HYBODONTIDAE Owen, 1846

*DURNONOVARIAODUS* gen. nov.

**LSID:** urn:lsid:zoobank.org:act:35DA49B9-B14E-4390-BEA3-9B99C9D9BAB0

**Diagnosis:** Hybodontiform shark-like chondrichthyan that is characterized by the following unique combination of morphological characters: palatoquadrate elongate with low and reduced palatobasal process and well-developed ethmoidal articular surface; Meckel’s cartilage elongate and rather deep with well-developed dental groove extending for approximately one-half its length of the Meckel’s cartilage; medial quadratomandibular joint on Meckel’s cartilage prominent and well-defined; articular cotylus on Meckel’s cartilage moderately well-developed and shallowly recessed; hyomandibular head formed into an anteriorly directed hook-like process; dorsal fin spines ornamented with strong, non-bifurcating costae; body covered by monodontode, thorn-like dermal denticles; dentition includes high-crowned multicuspid teeth that are symmetrical to slightly asymmetrical in labio-lingual view displaying disjunct monognathic heterodonty; tooth crown strongly labio-lingually flattened; main cusp high and fairly wide at its base without sigmoidal profile; main cusp flanked on each side by up to three pairs of low but well-developed lateral cusplets; cutting edges slightly labially displaced, continuous, and sharp without serrations; labial crown base is slightly incised above the crown-root junction and somewhat swollen; crown-root junction straight; lingual and labial crown faces ornamented with very short, inconspicuous vertical folds aligned along the base above the crown-root junction; tooth root prominent, about as high apico-basally as deep labio-lingually, and slightly lingually displaced beneath the tooth crown; basal root face flat with shallow depression extending along the labial edge; lingual and labial root face perforated by numerous small, densely arranged foramina and large, regularly arranged foramina that occur aligned along the bases; morphological variation passing posteriorly through the dentition encompasses distal inclination and reduction of principal cusp; anterior teeth symmetrical in labio-lingual view with moderately robust main cusp and divergent lateral cusplets; lateral teeth asymmetrical in labio-lingual aspect with wide, triangular-shaped and slightly distally inclined main cusp; posterior teeth asymmetrical and low in in labio-lingual view.

**Etymology:** The genus name is derived from Durnonovaria, the ancient name of the town of Dorchester from which the name Dorset derives, and the Greek noun *odus* (ὀδούς), meaning tooth.

**Type species:** *Durnonovariaodus maiseyi* sp. nov.

*DURNONOVARIAODUS MAISEYI* gen. et sp. nov.

(Figs. 2–6)

**Figure 2:**
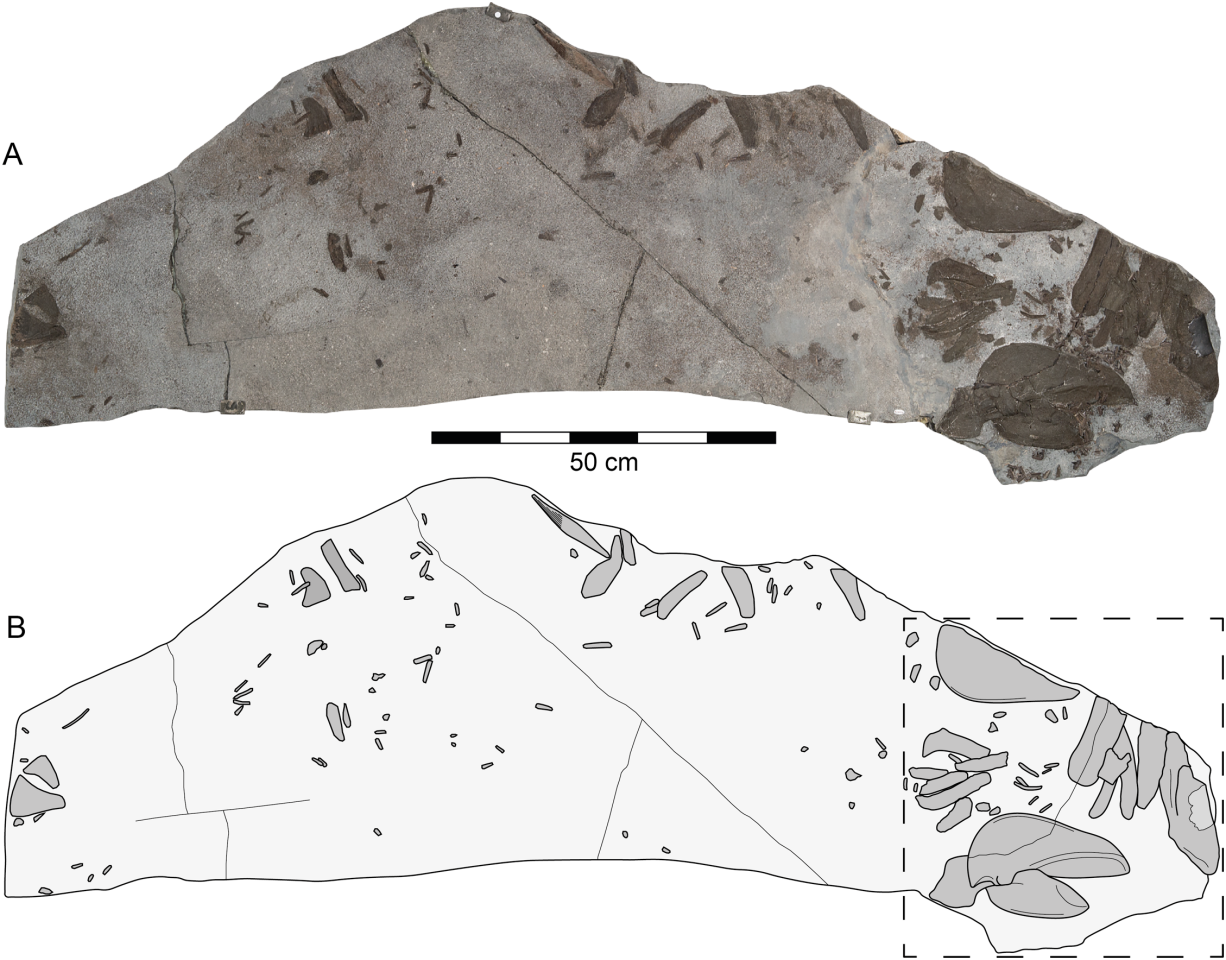
*Durnonovariaodus maiseyi* gen. et sp. nov., MJML K1624, holotype, from the Upper Jurassic Kimmeridge Clay Formation (early Tithonian) near Freshwater Steps, Encombe, Dorset, England. (A) Slab containing specimen. (B) Interpretative line drawing (dashed box indicates splanchnocranial elements shown in Fig. 3).

**Figure 3:**
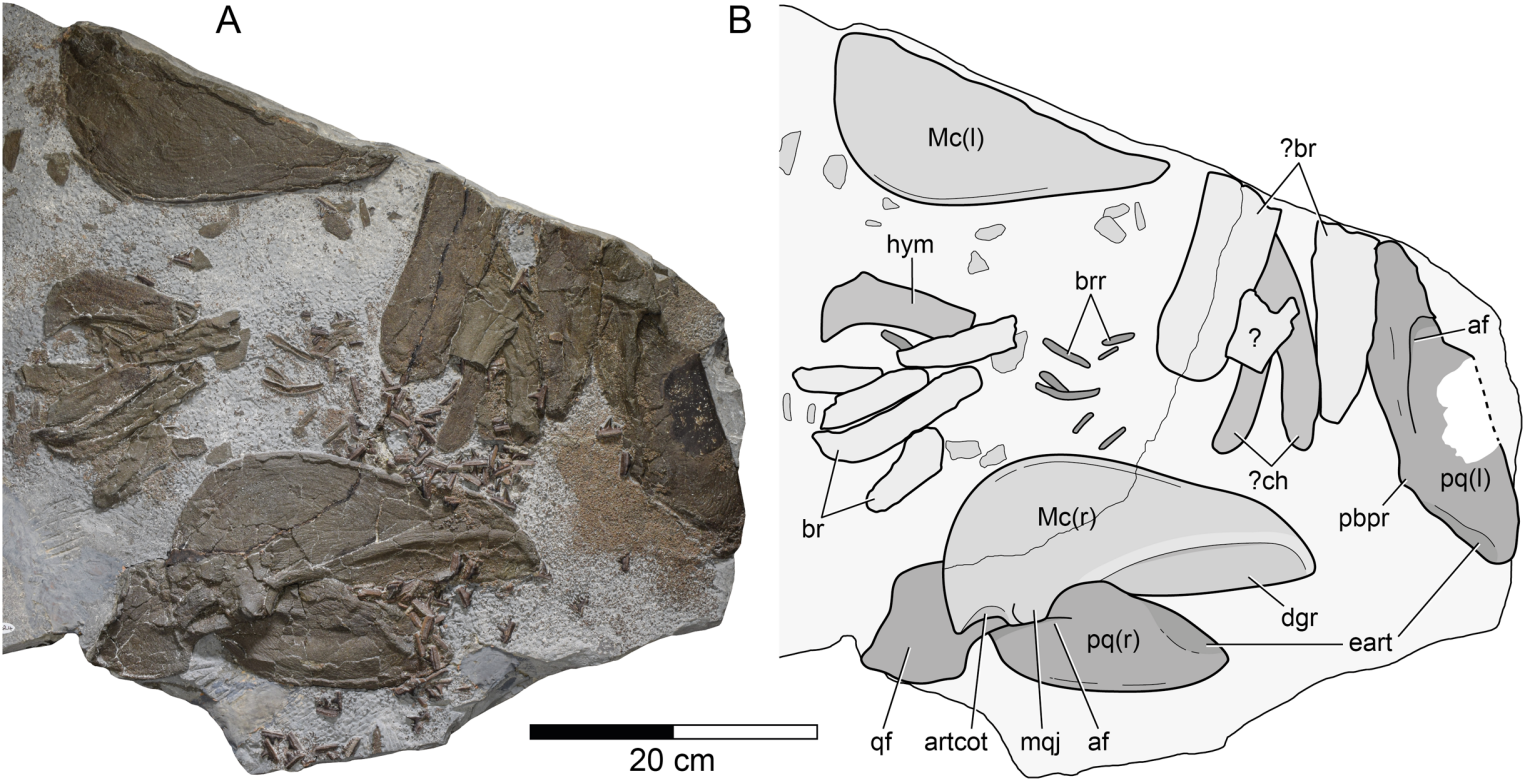
*Durnonovariaodus maiseyi* gen. et sp. nov., MJML K1624, holotype, from the Upper Jurassic Kimmeridge Clay Formation (early Tithonian) near Freshwater Steps, Encombe, Dorset, England. (A) Splanchnocranium; (B) Interpretative line drawing. Anatomical abbreviations: af, adductor fossa; artcot, articular cotylus; br, branchial element; brr, branchial rays; ch, ceratohyal; dgr, dental groove; eart, ethmoidal articulation; hym, hyomandibular; l, left (in parentheses); Mc, Meckel’s cartilage; mqj, medial quadratomandibular joint; pbpr, palatobasal process; pq, palatoquadrate; qf, quadrate flange; r, right (in parentheses).

**Figure 4:**
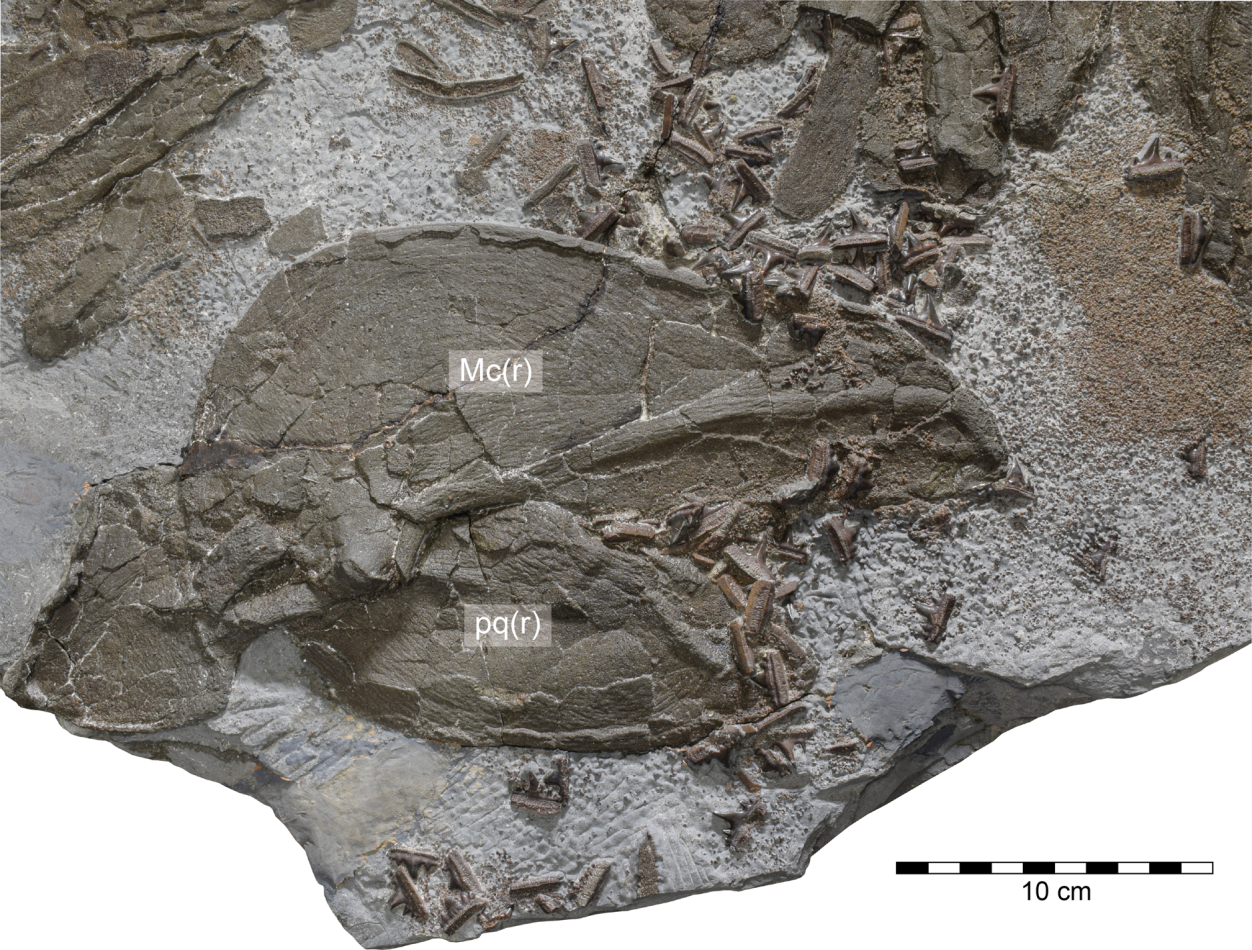
*Durnonovariaodus maiseyi* gen. et sp. nov., MJML K1624, holotype, from the Upper Jurassic Kimmeridge Clay Formation (early Tithonian) near Freshwater Steps, Encombe, Dorset, England. Overview of dentition (for anatomical abbreviations see caption to Fig. 3).

**Figure 5:**
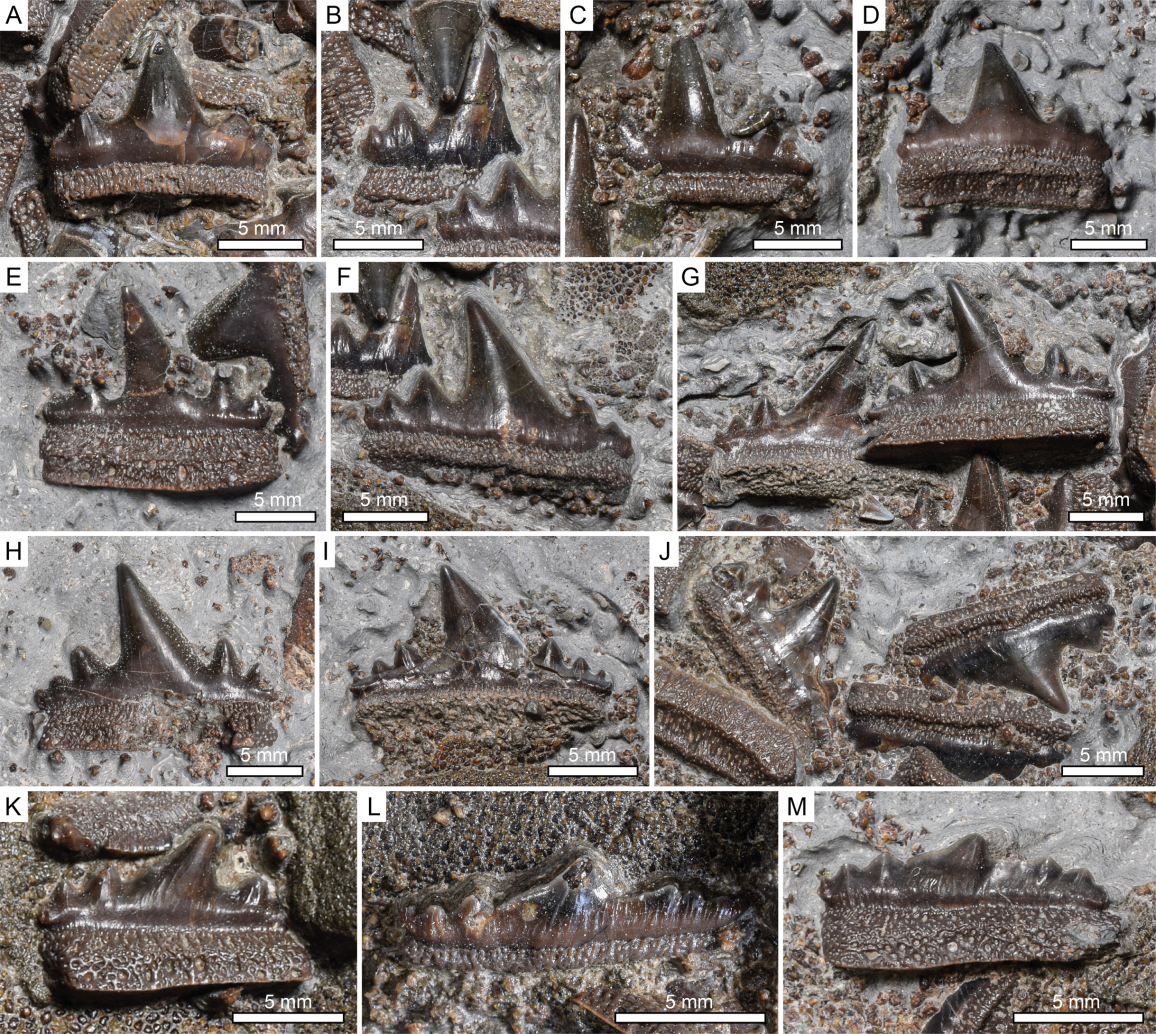
*Durnonovariaodus maiseyi* gen. et sp. nov., MJML K1624, holotype, from the Upper Jurassic Kimmeridge Clay Formation (early Tithonian) near Freshwater Steps, Encombe, Dorset, England. Close-up view of teeth. (A–D) Anterior teeth in labial views. (E) Antero-lateral tooth in lingual view. (F) Lateral tooth in labial view. (G–I) Lateral teeth in lingual aspects (J) Postero-lateral teeth in labial views (K) Postero-lateral tooth in lingual view. (L, M) Extreme posterior teeth in (L) labial and (M) lingual aspect.

**Figure 6:**
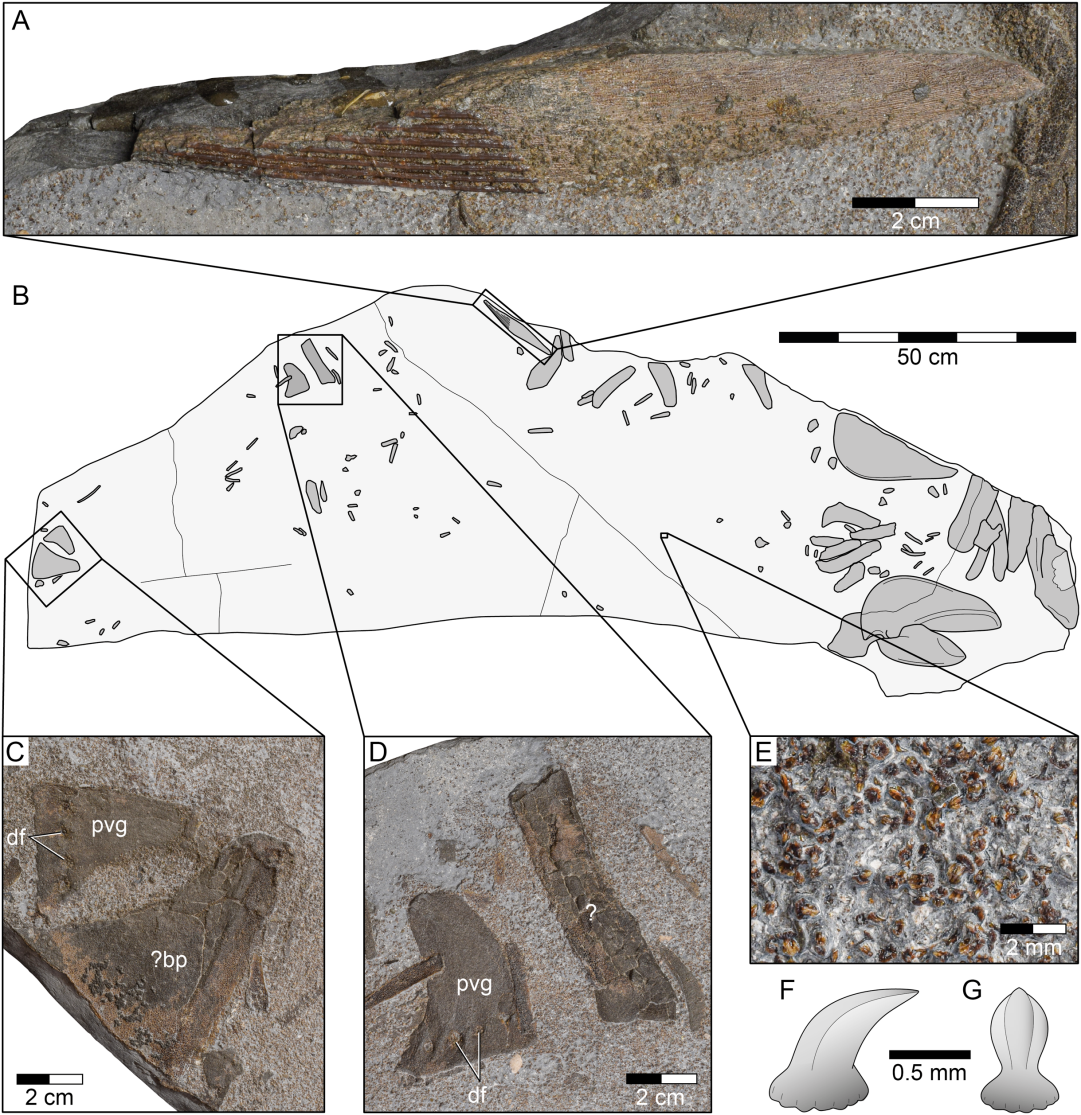
*Durnonovariaodus maiseyi* gen. et sp. nov., MJML K1624, holotype, from the Upper Jurassic Kimmeridge Clay Formation (early Tithonian) near Freshwater Steps, Encombe, Dorset, England. (A) Dorsal fin spine. (B) Interpretative line drawing of complete specimen. (C, D) Pelvic girdle. (E) Close-up view of dermal denticles. (F, G) Simplified sketch drawing of dermal denticle in (F) lateral and (G) anterior view. Anatomical abbreviations: bp, basal plate; df, diazonal nerve foramina; pvg, pelvic half-girdle.

2020 *Planohybodus peterboroughensis* Rees and Underwood; Underwood, text-fig. 2.3A.

**LSID:** urn:lsid:zoobank.org:act:2E9EAEC3-16FA-4304-A6FE-1123135A46BF

**Diagnosis:** As for genus (by monotypy).

**Holotype:** MJML K1624, a slab of rock preserving disarticulated elements of the splanchnocranium with associated teeth, numerous dermal denticles, a single fragmentary dorsal fin spine, and the pelvic girdle, plus abundant cartilage fragments of uncertain identity.

**Type locality and horizon:** Freshwater Steps, Encombe, Dorset, England; Upper Kimmeridge Clay Formation, *Pectinatites pectinatus* ammonite zone, early Tithonian, Late Jurassic.

**Etymology:** Species named in honour of John G. Maisey for his significant work on better understanding hybodontiform taxonomy and systematics and his contribution to the field of palaeoichthyology in general.

**Description**

The holotype and only specimen of *Durnonovariaodus maiseyi* gen. et sp. nov., MJML K1624, is preserved on a slab of rock of about 1,785 mm maximum length and 700 mm maximum width preserving disarticulated elements of the splanchnocranium with associated teeth, a single fragmentary dorsal fin spine, the pelvic girdle, as well as abundant cartilage fragments of uncertain identity, plus countless dermal denticles scattered all across the slab (Fig. 2). The endoskeletal remains are strongly compressed, but still show a certain degree of relief suitable for identifying morphological features. They are composed of well-mineralized, tessellated cartilage, which gives them a rough and scratchy surface texture. The scattered, but closely arranged skeletal elements support our interpretation that all belong to a single specimen.

**Splanchnocranium.** The splanchnocranium is incomplete and highly disarticulated. It includes the mandibular arch as well as part of the hyoid arch and gill arches (Fig. 3).

The mandibular arch is disarticulated and includes the paired palatoquadrates and Meckel’s cartilages. The right palatoquadrate and the right Meckel’s cartilage are complete, while their left counterparts are incomplete.

The right palatoquadrate is visible in lateral aspect, measuring 257 mm in maximum length and 89 mm in height. The left palatoquadrate is less well-preserved and exposed in lateral view. It is incomplete in its most-posterior portion and along its ventral margin. The right Meckel’s cartilage is exposed in medial view, measuring 261 mm in maximum length and 125 mm in maximum height. Its left counterpart is visible in lateral aspect and lacks its dorsal portion.

The palatoquadrate of *Durnonovariaodus maiseyi* gen. et sp. nov. is elongate and rather massive. It can be roughly divided into an anterior palatine and a posterior quadrate portion. The latter is formed into a large, well-developed quadrate flange, which anteriorly gives rise to a prominent, well-defined ridge that bounds a deep adductor fossa dorsally. The dorsal margin of the palatoquadrate exhibits a low and reduced palatobasal process, which is located at about one-third the total palatoquadrate length from the anterior tip. The palatoquadrate is widely convex antero-dorsally and slopes slightly downwards towards its anterior tip. A large articulation surface for the ethmoid process of the neurocranium extends along the antero-dorsal margin of the palatoquadrate. The anterior tip of the palatoquadrate is bluntly pointed and the ventral margin of the anterior palatine portion of the palatoquadrate is widely convex and reinforced by a narrow, slightly elevated ridge.

The Meckel’s cartilage is elongate, rather deep posteriorly and tapers slightly towards its anterior tip, which is bluntly pointed rather than sharply tipped. Medially, there is a deep, well-developed dental groove, which extends approximately one-half the length of the Meckel’s cartilage. Ventrally, the dental groove is delimited by a prominent ridge. The dorsal margin of the Meckel’s cartilage is straight for the length of the accompanying dental groove until it forms a wide and low indentation that is delimited posteriorly by a large, well-defined medial quadratomandibular joint. The articular cotylus for the articulation with the palatoquadrate is moderately well-developed and shallowly recessed. A lateral quadratomandibular joint could not be observed. The postero-ventral margin of the Meckel’s cartilage is widely convex and merges smoothly into the ventral margin, which is straight for most of its length and reinforced by a narrow, slightly elevated ridge. Laterally, the Meckel’s cartilage bears a similarly developed ridge extending along its ventral margin. Labial cartilages could not be identified.

The hyoid arch and gill arches of *Durnonovariaodus maiseyi* gen. et sp. nov. are incomplete and very badly preserved. There is a single hyomandibular and two slender, slightly curved cartilages that are here tentatively identified as the ceratohyals, plus numerous well-calcified cartilage fragments of uncertain identity. The preserved hyomandibular is broken distally and could not be identified as either left or right. The proximal end of the hyomandibular, which articulates with the neurocranium, is formed into an anteriorly directed hook-like process.

**Dentition.** The holotype of *Durnonovariaodus maiseyi* gen. et sp. nov. comprises about 80 disarticulated teeth that are scattered on and around the right Meckel’s cartilage and right palatoquadrate (Fig. 4), suggesting that they derive from both the upper and lower dentition. Morphologically, the teeth can be differentiated into those coming from tooth files of anterior, lateral and posterior positions (see below), indicating a disjunct monognathic heterodonty. There is no indication for dignathic heterodonty in *Durnonovariaodus maiseyi* gen. et sp. nov., but this must be considered as tentative due to the incomplete and disarticulated nature of the holotype specimen, pending the discovery of more complete material.

The dentition of *Durnonovariaodus maiseyi* gen. et sp. nov. encompasses relatively large, up to 18 mm wide and 12 mm high, symmetrical to slightly asymmetrical multicuspid teeth that are characterized by strongly labio-lingually flattened crowns displaying a high, fairly wide and pointed main cusp without a sigmoidal profile (Fig. 5). The main cusp is usually flanked by two to three pairs of low but well-developed lateral cusplets, which diminish in size away from the main cusp and reach up to one-third its height. The cutting edges are slightly labially displaced, sharp and continuous, extending from the principal cusp across all lateral cuplets. There are no serrations on the cutting edges. The labial crown base is slightly incised above the crown-root junction and somewhat swollen. The tooth crown ornamentation is reduced and comprises very short, inconspicuous vertical folds that occur on both the lingual and labial bases of the crown above the crown-root junction. The distribution of these vertical folds slightly differs on the lingual and labial faces, with those occurring on the lingual face occasionally being restricted to the bases below the lateral cusplets only, or may even be absent entirely. The crown-root junction is straight lingually and labially.

The tooth root is prominent, about as high apico-basally as deep labio-lingually, and slightly lingually displaced beneath the crown, forming a narrow, lingually sloping shelf. The basal root face is flat and bears a shallow depression that extends along the labial edge. The lingual and labial faces of the root are perforated by numerous tiny, densely arranged foramina, resulting in a somewhat trabecular appearance of the root. In addition, a series of larger, rather regularly arranged foramina occurs along both the lingual and labial base of the root.

The morphological variation that passes posteriorly through the dentition of *Durnonovariaodus maiseyi* gen. et sp. nov. mainly involves a distal inclination and reduction of the principal cusp. Teeth from anterior positions are symmetrical and display a moderately robust and erect principal cusp that is flanked by two pairs of low, slightly divergent lateral cusplets (Figs. 5A–5C). These are, as measured from the crown-root junction, up to one-half the height of the crown. In addition, a third pair of very small to incipient lateral cusplets may be developed (Fig. 5D).

Lateral teeth exhibit a fairly wide, triangular-shaped and slightly distally inclined main cusp, which has a more or less straight mesial but a slightly concave distal cutting edge (Figs. 5E–5I). The main cusp is usually flanked on each side by three pairs of lateral cusplets. These are up to one-half the height of the crown as in teeth of anterior positions.

Posterior teeth have a wide and very low profile. The main cusp is wide, particularly low and distally inclined and has a long, slightly undulating mesial cutting edge (Figs. 5J–5M). It is flanked on each side by two to three pairs of low, commonly reduced lateral cusplets.

**Dorsal fin spine.** The holotype of *Durnonovariaodus maiseyi* gen. et sp. nov. includes a single dorsal fin spine only (Figs. 6A, 6B). The fin spine is incomplete and exposed in left lateral view, lacking its distal portion. It is ornamented with strong, non-bifurcating costae. The unornamented fin spine base, which includes the deeply inserted posterior slot that received the cartilaginous basal plate of the dorsal fin, appears to have been rather long. No further information can be retrieved due to the poor state of preservation of the dorsal fin spine. There is a cartilage fragment of roughly triangular shape, which may represent a dorsal basal fin plate (Figs. 6B, 6C).

**Pelvic girdle.** The pelvic girdle of *Durnonovariaodus maiseyi* gen. et sp. nov. is represented by two separate pelvic half-girdles, both displaying a series of diazonal nerve foramina aligned along the distal margin (Figs. 6B–6D). There is an elongate, broken cartilage preserved in close proximity to one of the pelvic girdle halves (Figs. 6B, 6D), whose precise identity remains unknown due to preservation.

**Dermal denticles.** The holotype of *Durnonovariaodus maiseyi* gen. et sp. nov. encompasses countless very small, densely packed dermal denticles that occur all across the bedding plane (Fig. 6E). Morphologically, the dermal denticles correspond to the ‘non-growing’ (monodontode) type. They all have a thorn-like appearance, measuring less than 1 mm in maximum height, with a circular to oval base and an upright, slightly recurved cusp displaying a few strong vertical folds that extend from the apex to the base of the cusp (Figs. 6F, 6G). These folds usually merge apically to form a keel-like leading edge extending along the anterior face of the cusp.

## Discussion

### Comparison

In the following, detailed comparisons between *Durnonovariaodus maiseyi* gen. et sp. nov. and other hybodontiforms is drawn. While the first section focuses on comparing and contrasting the dentition of *Durnonovariaodus maiseyi* gen. et sp. nov. with that of other hybodontiforms, particularly those that are known to have developed teeth of similar morphologies, the second section addresses similarities and differences in skeletal anatomy between the new taxon and better known hybodontiforms. Note that the genus *Asteracanthus* Agassiz, 1837 is here considered distinct from *Strophodus* Agassiz, 1838, following Stumpf et al. (2021). In addition, the systematic position of *Durnonovariaodus maiseyi* gen. et sp. nov. within Hybodontiformes is discussed in the light of currently available hypotheses of their interrelationships.

Specimen MJML K1624, which is here designated as new genus and species, *Durnonovariaodus maiseyi* gen. et sp. nov., was briefly described by Underwood (2020), who referred it to *Planohybodus peterboroughensis* Rees & Underwood, 2008, which is known from rare dental and fragmentary skeletal material from the Callovian–Oxfordian of England. Teeth of *P*. *peterboroughensis*, although morphologically similar, are readily distinguished from those of *Durnonovariaodus maiseyi* gen. et sp. nov., by possessing fairly symmetrical, more strongly ornamented crowns with a higher and more slender central cusp, which is flanked by two or three pairs of lateral cusplets that are up to one-quarter the height of the of the central cusp. Furthermore, unlike in *Durnonovariaodus maiseyi* gen. et sp. nov., the dentition of *P*. *peterboroughensis* appears to have been characterized by a gradual rather than disjunct monognathic heterodonty, as expressed by variations in height and width of the main cusp and by a weak dignathic heterodonty, with teeth of the lower jaw being narrower and less heavily ornamented, exhibiting a rather gracile main cusp, which may be slightly mesio-distally expanded at mid-height, plus smaller, less well-developed lateral cusplets (Rees & Underwood, 2008).

The remaining species currently placed in *Planohybodus* are known from isolated teeth only and include *P*. *grossiconus* (Agassiz, 1833–1844) from the Bathonian of England, Scotland and France (Woodward, 1889; Rees & Underwood, 2006, 2008) and *P*. *ensis* (Woodward, 1916) from the Berriasian–Barremian of England and Spain (Patterson, 1966; Underwood & Rees, 2002; Bermúdez-Rochas, 2009; Duffin & Sweetman, 2011; Turmine-Juhel et al., 2019). Additionally, poorly preserved teeth from the Berriasian of Bornholm, Denmark, may present an as yet undescribed species of *Planohybodus* (Rees, 2001; Rees & Underwood, 2008).

The species *P*. *marki* Pinheiro et al., 2013 from the pre-Aptian Early Cretaceous of Brazil, which is represented by a few fragmentary tooth crowns, is here regarded as *nomen dubium* due to the poor state of preservation and the absence of any dental features that would unambiguously support its inclusion in the genus *Planohybodus*.

Teeth of *P*. *grossiconus* and *P*. *ensis* are very similar to those of *P*. *peterboroughensis*, which makes species identification of isolated tooth crowns difficult, particularly because dental characters for use in differentiation between these three species mainly relate to differences in main cusp proportions and the number of lateral cusplets, besides minor variations in ornamentation (Rees & Underwood, 2008). In addition, faint serrations may be developed on the cutting edges in larger teeth of *P*. *ensis*, unlike in *P*. *peterboroughensis* and *P*. *grossiconus* (Underwood & Rees, 2002; Bermúdez-Rochas, 2009) as well as *Durnonovariaodus maiseyi* gen. et sp. nov.

High-crowned, labio-lingually flattened dental morphologies with partly serrated cutting edges also characterize teeth of *Secarodus*, which was erected by Rees & Underwood (2008) for distinctive teeth from the Bathonian of England that were originally described as *Hybodus polyprion* by Agassiz (1843). However, teeth of *Secarodus* differ from those of *Planohybodus ensis* (and *Planohybodus* in general) in exhibiting lower crowns with a fairly wide, triangular-shaped main cusp and in possessing cutting edges characterized by more strongly developed serrations. In addition, the dentition of *Secarodus* is characterized by a disjunct monognathic heterodonty, with anterior teeth being almost symmetrical in profile, a condition clearly separating them from teeth of lateral and posterior positions. A quite similar heterodonty pattern characterizes the dentition of *Durnonovariaodus* gen. nov., although lateral teeth of the latter are less asymmetrical in profile as compared to those of *Secarodus*. The ornamentation is reduced in teeth of *Secarodus* and consists of short vertical folds along the base of the crown, resembling the condition in *Durnonovariaodus* gen. nov., which otherwise lacks a small, knob-like protuberance at the base of the labial crown face, a feature that has been found to occasionally occur in teeth of *Secarodus*. The main character separating teeth of *Secarodus* from those *Durnonovariaodus* gen. nov. is the presence of weak to moderately well-developed serrations occurring on the lower mesial cutting edges of the main cusp and cusplets.

The presence of high-crowned, strongly labio-lingually compressed multicuspid teeth with fully serrated cutting edges characterizes teeth of *Priohybodus arambourgi* d’Erasmo, 1960 from the Kimmeridgian–Hauterivian/Barremian of Africa, Yemen and Uruguay (Tabaste, 1963; Goodwin et al., 1999; Duffin, 2001; Cuny et al., 2004; Soto, Perea & Toriño, 2012). However, unlike in *Secarodus*, the dentition of *Priohybodus* is rather homodont to include close to symmetrical teeth with a prominent principal cusp and up to five pairs of strongly divergent lateral cusplets, suggesting a closer phylogenetic relationship with *Planohybodus* than with *Secarodus* and other hybodontiforms (Rees & Underwood, 2008; Soto, Perea & Toriño, 2012).

The remaining hybodontiforms that have developed teeth with fully serrated cutting edges are *Pororhiza molimbaensis* Casier, 1969 from the Albian of Congo, *Thaiodus ruchae* Cappetta, Buffetaut & Suteethorn, 1990 from the Aptian–Albian of Thailand, Tibet and China (Cappetta et al., 2006; Cuny et al., 2008; Mo et al., 2016), and *Mukdahanodus trisivakulii* Cuny, Cavin & Suteethorn, 2009 from the pre-Aptian Early Cretaceous of Thailand. In addition, *Mukdahanodus* is also represented by a possible second species from the Barremian–Aptian of Malaysia (Teng et al., 2019). All of these species are characterized by quite uniquely shaped teeth with distinctively low crowns. A main cusp is either absent in these species or it is very low and blunt.

*Durnonovariaodus* gen. nov. has costate dorsal fin spines, a condition shared with most other hybodontiforms (Maisey, 1978), except for *Asteracanthus* and *Strophodus*, which currently are the only known hybodontiforms for which dorsal fin spines with an ornamentation consisting of small to moderately well-developed, more or less regularly arranged tubercles can be unambiguously be confirmed (Stumpf et al., 2021), although this feature certainly was more widely distributed among hybodontiforms (cf. e.g., Werner, 1989; Case & Cappetta, 2004; Underwood & Cumbaa, 2010; Cicimurri, Ciampalgio & Runyon, 2014). *Planohybodus*, which shares with *Durnonovariaodus* gen. nov. the presence of costate dorsal fin spines, seems to be differentiated from the latter in having fin spines with proximally bifurcating costae, as inferred from fin spine material referred to the *Planohybodus* type species, *P*. *peterboroughensis* (Rees & Underwood, 2008). However, the phylogenetic significance of this difference in fin spine ornamentation needs to be tested.

The countless small thorn-like dermal denticles present in the holotype of *Durnonovariaodus maiseyi* gen. et sp. nov., which would certainly have covered the body in life, closely resemble those covering the body of *Hamiltonichthys mapesi* Maisey, 1989 and those occurring on top of the head of *Egertonodus basanus* (Egerton, 1845) and *Hybodus delabechei* Charlesworth, 1839 (Reif, 1978; Maisey, 1983, 1989; note that the generic identity of *H*. *delabechei* remains unresolved, see Rees, 1998; Maisch & Matzke, 2016). Dermal denticles of quite similar morphology cover the body of *Hybodus fraasi* Brown, 1900, a species tentatively referred to *Egertonodus* by Maisey (1987). Dermal denticles of this species, however, differ from those of the aforementioned taxa in having a larger base that is ovoid in outline carrying a rather low, more strongly compressed cusp that is formed into a blade-like keel (Maisey, 1986; Thies & Leidner, 2011). The shagreen covering the body of *Tribodus limae* Brito & Ferreira, 1989 is composed of similarly developed denticles, but also includes smaller denticles of different morphology, which co-occur intercalated between the larger ones, resulting in a unique two-size squamation pattern otherwise unknown in hybodontiforms (Maisey & Denton, 2016). Dermal denticles of *Asteracanthus ornatissimus* have a circular base carrying an upright, cone-like cusp that exhibits numerous vertical folds that radiate from the apex to the base of the cusp (Stumpf et al., 2021), resembling those covering the head of *Planohybodus peterboroughensis* (Rees & Underwood, 2008). In addition, similarly developed denticles have been observed in the snout region of *Hamiltonichthys* (Maisey, 1989). In ‘*Hybodus*’ *delabechei*, cone-like dermal denticles are present on both the lower jaws and on the roof of the mouth cavity, with the latter co-occurring with dermal denticles that correspond to the ‘growing’ (polydontode) type (Reif, 1978). Despite being absent in the holotype specimen of *Durnonovariaodus maiseyi* gen. et sp. nov., growing dermal denticles actually may have been present in life, because this type of dermal denticles is likely to have been restricted to the oropharyngeal region in at least some, if not all, hybodontiforms (Maisey & Denton, 2016).

Morphologically, the palatoquadrate of *Durnonovariaodus* gen. nov. closely resembles that of *Egertonodus* and *Hybodus*, particularly in having a distinct, antero-dorsally positioned articulation surface for the ethmoid process of the neurocranium and forming a palatobasal process that projects dorsally from the palatine moiety (Maisey, 1982, 1989). The jaw suspension of *Durnonovariaodus* gen. nov. is therefore likely to have been hyostylic (sensu Lane & Maisey, 2012). This type of jaw suspension may have also been present in *Asteracanthus*, whose palatoquadrates otherwise lack a palatobasal process and instead display a deeply recessed dorso-medial articulation facet, presumably for the articulation with the ectethmoid process of the neurocranium (Stumpf et al., 2021). *Tribodus* is unique among hybodontiforms in having a jaw suspension reminiscent of the euhyostylic condition present in modern batomorphs (Maisey & de Carvalho, 1997; Lane & Maisey, 2012). Its palatoquadrates are short, transversally oriented and connected symphyseally but not fused, lacking any direct articulation with the neurocranium, although there may have been ligamentous connections between the palatoquadrates and the neurocranium, probably homologous to the palatobasal articulation present in most other hybodontiforms (Lane & Maisey, 2012).

The Meckel’s cartilage of *Durnonovariaodus* gen. nov. is reminiscent of that of *Hybodus*, *Egertonodus* and *Asteracanthus* in displaying an elongate and comparably low profile (Maisey, 1982, 1983, 1987; Stumpf et al., 2021). This distinguishes it from other hybodontiforms like *Palaeobates* Meyer, 1849, *Acrodus* Agassiz, 1837 and *Crassodus* Maisch & Matzke, 2016, which have relatively massive and deep Meckel’s cartilages (Maisey, 1982; Romano & Brinkmann, 2010; Lane & Maisey, 2012; Maisch & Matzke, 2016). The antero-posterior dimension displayed by the dental groove, which extends about one-half the length of the Meckel’s cartilage, is similar to that observed in other hybodontiforms (Fraas, 1896; Maisey, 1983, 1987; Romano & Brinkmann, 2010; Lane & Maisey, 2012) and many other chondrichthyan outgroup taxa basal to hybodontiforms (e.g., Hotton, 1952; Coates & Sequeira, 2001; Long et al., 2015).

*Durnonovariaodus* gen. nov. shares with *Hybodus* and *Egertonodus* a hyomandibular with an elongate, slightly anteriorly directed proximal end (Maisey, 1982, 1983). This contrasts with *Tribodus*, in which the hyomandibular is rather short and more rod-like, lacking an enlarged proximal end (Lane & Maisey, 2012).

**Figure 7:**
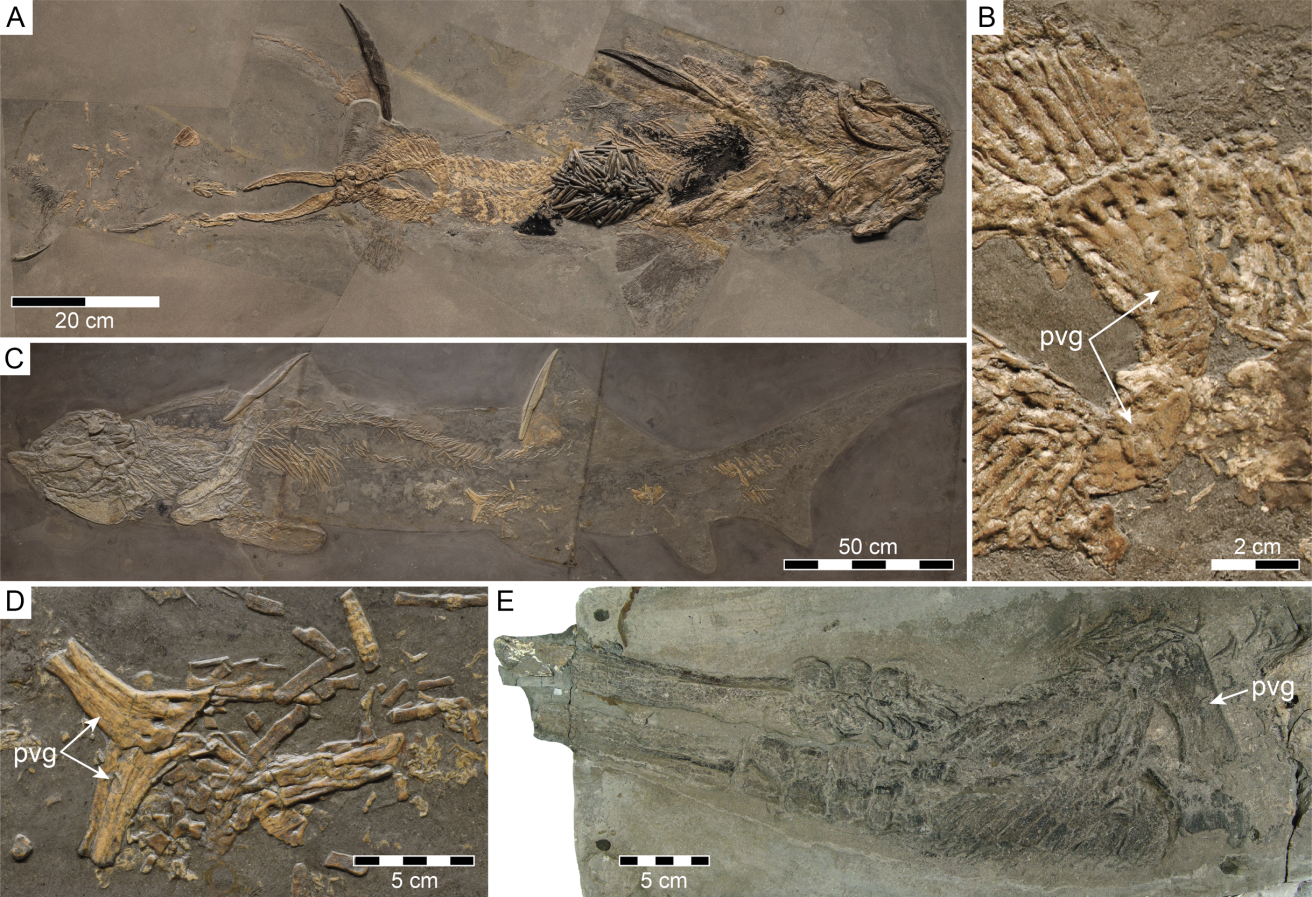
Separate pelvic half-girdles (pvg) in hybodontiforms. (A, B) Male specimen of *Hybodus hauffianus* Fraas, 1895, SMNS 10060, from the Lower Jurassic Posidonienschiefer Formation of Holzmaden, Baden-Württemberg, Germany, in (A) total view and (B) close-up view of pelvic girdle. (C, D) Female specimen of *H*. *hauffianus*, SMNS 15150, from the Posidonienschiefer Formation of Holzmaden in (C) total view and (D) close-up view of pelvic girdle. (E) Hybodontiformes gen. et sp. indet., NHMUK PV P 339, from the Lower Jurassic of Lyme Regis, Dorset, England, showing pelvic girdle and associated clasper complex.

The holotype specimen of *Durnonovariaodus* gen. nov. preserves two separate pelvic half-girdles, a condition present in *Tristychius* Agassiz, 1837 (Dick, 1978) and more basal chondrichthyans (e.g., Zangerl & Case, 1976; Dick, 1981; Lund, 1985). In elasmobranchs, the paired halves of the pelvic girdle are fused to form a continuous puboischiadic bar, a feature considered by Compagno (1973, 1977) to be a synapomorphy separating them from more basal chondrichthyan outgroups. This view, however, was refuted by Maisey (1989) based on the presence of a continuous puboischiadic bar occurring in males of the apparently primitive hybodontiform *Hamiltonichthys*. A puboischiadic bar is otherwise absent in females of the same genus, which retain the plesiomorphic condition of separate pelvic half-girdles. Maisey (1989) regarded this intraspecific variation in pelvic girdle morphology as an evolutionary primitive character state separating *Hamiltonichthys* from more derived hybodontiforms, which he suggested to have developed independently of their sex a continuous puboischiadic bar. However, direct fossil support for this hypothesis is still lacking due to the limited number of sufficiently and well-preserved fossil material. In fact, currently available information on the pelvic girdle morphology of hybodontiforms other than *Hamiltonichthys* is limited to males of *Lissodus* and *Hybodus* only, more precisely to males of *Lissodus cassangensis* (Teixeira, 1956) from the Lower Triassic of Angola and *Hybodus hauffianus* Fraas, 1895 from the Lower Jurassic of Germany, which are traditionally accepted to have developed a puboischiadic bar (Brown, 1900; Maisey, 1982; Antunes et al., 1990). However, a re-investigation of the specimen on which the presence of a puboischiadic bar in males of *Hybodus hauffianus* was initially claimed (Fig. 7A), actually revealed the possession of two separate pelvic half-girdles, which are in part covered by an amorphous, light-brown phosphatic mass that presents preserved part of the stomach contents (see Fig. 7B; cf. Brown, 1900: pl. 16, fig. 1.; Maisey, 1982: text-fig. 13B). This feature is also shared by female individuals of *H*. *hauffianus* (Figs. 7C, 7D; see also Hauff et al., 2014: fig. 190), and may in fact have been more widely distributed among supposedly more advanced hybodontiforms than previously thought, as also suggested by a large indeterminate hybodontiform from the Lower Jurassic of Lyme Regis, England, preserving an incomplete clasper complex associated with a single half-girdle (Fig. 7E). In addition, according to Maisey (1982), males of *Lissodus cassangensis* possess a puboischiadic bar, although the presence of this feature cannot unambiguously be attested for either males or females of this species due to the poor preservation of the available material (Maisey, 1982; Antunes et al., 1990). In fact, as judged by the interpretative drawing of the pelvic fin and clasper complex of *L*. *cassangensis* provided by Maisey (1982: text-fig. 13A), males of this species actually appear to have developed two separate pelvic half-girdles, which Maisey (1982: 25) interpreted as a preservation artefact due to “superposition of one fin on the other”, but more complete material is needed to confirm or refute Maisey’s interpretation.

In summary, males of *Hamiltonichthys* are as yet the only known hybodontiforms for which the presence of a puboischiadic bar can unambiguously be confirmed. This contrasts with females of *Hamiltonichthys*, which together with both males and females of *Hybodus* retain the evolutionary primitive condition of two separate pelvic half-girdles otherwise present in chondrichthyan outgroups basal to Hybodontiformes. Whether the possession of two separate pelvic half-girdles is present in individuals of both sexes of *Durnonovariaodus* gen. nov. or related to sex-specific variation in pelvic girdle morphology as in *Hamiltonichthys* nevertheless remains impossible to determine without having more complete material.

### Systematic affinities

*Durnonovariaodus maiseyi* gen. et sp. nov. is characterized by a unique combination of dental characters, indicating close architectural similarities to *Secarodus polyprion*, whose familial affinities still remain ambiguous and unresolved. When initially described, *Secarodus* was placed together with *Planohybodus* in Hybodontinae by Rees & Underwood (2008) based on the presence of high-crowned multicuspid teeth, following Maisey (1989) who included this subfamily together with Acrodontinae in the family Hybodontidae. The grouping proposed by Maisey (1989) is mainly based on the presence of an osteodont tooth histotype, which he considered as a derived feature among hybodontiforms. On the contrary, genera with low-crowned teeth possessing the orthodont tooth histotype were regarded by Maisey (1989) to form an assemblage of phylogenetically plesiomorphic hybodontiforms, except for the supposed durophagous genus *Palaeobates*, which he tentatively referred to Hybodontidae, particularly due to the presence of cephalic spines with a T-shaped basal plate as well as some cranial features shared with species included in Hybodontinae and Acrodontinae. Although generally accepted, this classification scheme still remains open to question, mainly because tooth histology patterns in hybodontiforms are more heterogenous and diverse than previously thought (e.g., Rees, 2001; Blażekowski, 2004; Stumpf et al., 2021), which makes a conclusive assessment of hybodontiform interrelationships as inferred from tooth histologies impossible based on the current data available, pending further research.

Rees (2008) proposed a phylogenetic hypothesis that placed *Secarodus* together with *Priohybodus* and *Planohybodus* in an unnamed hybodontid subfamily (informally referred to as “priohybodontines” by Soto, Perea & Toriño, 2012) based on the shared presence of strongly labio-lingually compressed, high-crowned multicuspid teeth with serrated cutting edges to form the sister group to Hybodontinae comprising *Hybodus* and *Egertonodus*, which both share high-crowned multicuspid grasping teeth with a slender main cusp that is close to circular in cross-section. However, the monophyletic grouping of *Secarodus*, *Priohybodus* and *Planohybodus* proposed by Rees (2008) may not be a natural one, because teeth of *Planohybodus* are in fact rather more similar to those of *Egertonodus* and *Hybodus* than to those of *Secarodus* and *Priohybodus* (Bermúdez-Rochas, 2009; Duffin & Sweetman, 2011; Turmine-Juhel et al., 2019), except for the presence of serrated cutting edges, a feature that is otherwise known to occur only rarely in teeth of *Planohybodus*. Further differences that may argue against the phylogenetic ties proposed by Rees (2008) relate to differences in heterodonty. In fact, the high degree of overlap in both dental morphology and heterodonty between *Secarodus* and *Durnonovariaodus* gen. nov. may suggest that both genera have formed a discrete monophyletic group of Mesozoic hybodontiforms characterized by uniquely shaped high-crowned multicuspid teeth. In consequence, one could argue that the dental traits shared by *Secarodus* and *Durnonovariaodus* gen. nov. are sufficient enough to justify the introduction of a new hybodontid subfamily for these two genera. However, dental morphology alone may not necessarily mirror evolutionary relationships of hybodontiforms, because some of them may have convergently evolved similar dentitions, as inferred from puzzling skeletal characteristics displayed by *Durnonovariaodus* gen. nov. and other apparently closely related hybodontiforms. For instance, *Durnonovariaodus* gen. nov. shares with *Hybodus* and *Egertonodus* a palatoquadrate with a palatobasal process and an ethmoidal articular surface, contrasting with *Asteracanthus*, which lacks a palatobasal process, but otherwise has teeth reminiscent of *Hybodus* and *Egertonodus* (Stumpf et al., 2021). Dorsal fin spines of *Asteracanthus* are ornamented with tubercles as opposed to costae present in fin spines of *Durnonovariaodus* gen. nov., *Hybodus, Egertonodus* and *Planohybodus*. In addition, cephalic spines of *Asteracanthus* have a uniquely shaped basal plate forming a robust posterior lobe and short lateral lobes. This differs to *Hybodus*, *Egertonodus*, and *Planohybodus*, which possess cephalic spines with a less robust, somewhat T-shaped basal plate (Maisey, 1983, 1987; Duffin, 1997; Rees & Underwood, 2008). On the other hand, *Egertonodus* has a single pair of cephalic spines (Maisey, 1983), while *Hybodus*, *Planohybodus* and *Asteracanthus* have a double pair of cephalic spines (Maisey, 1987; Rees & Underwood, 2008; Stumpf et al., 2021). The phylogenetic importance of all these differences, however, still remains unclear and needs to be tested. Likewise, much uncertainty still surrounds the variation in pelvic girdle morphology among hybodontiforms, pending the discovery of more complete skeletal material.

Consequently, given all these inconsistencies, we tentatively recommend referring *Durnonovariaodus* gen. nov. to Hybodontidae, particularly due to the close skeletal similarities shared with *Hybodus* and *Egertonodus*. However, further research is needed, pending a detailed re-evaluation of hybodontiform tooth mineralization patterns, combined with a subsequent phylogenetic analysis utilizing robust cladistic principles. However, resolving these issues is beyond the scope of the present study and will be published elsewhere.

### Paleoecology

*Durnonovariaodus* gen. nov. was certainly among the larger hybodontiforms, probably reaching a maximum length of up two meters, as inferred from size comparisons with taxa for which more complete skeletal material is available (cf. Fig. 7A; see also Koken, 1907; Urlichs, Wild & Ziegler, 1994; Stumpf et al., 2021), thus making it one of the largest chondrichthyans to have ever roamed the Jurassic seas.

The Jurassic represents an important period in the evolutionary history of chondrichthyans, because it was the time when crown group elasmobranchs comprising sharks, skates and rays underwent their first major radiations, resulting in profound faunal turnover events among chondrichthyan communities (Underwood, 2006; Kriwet, Kiesling & Klug, 2009; Guinot & Cavin, 2016, 2020; Stumpf & Kriwet, 2019). By the Late Jurassic, crown group elasmobranchs had become taxonomically diverse and geographically widespread forming the most dominant chondrichthyan group (e.g., Underwood, 2002; Kriwet & Klug, 2004, 2008; Szabó, 2020), suggesting an increasing risk of niche overlap with hybodontiforms. However, despite the apparently high competition potential with their more advanced chondrichthyan counterparts, Late Jurassic hybodontiforms may have avoided direct competition by exploiting different food resources, as suggested by differences in body size. While sharks, rays and skates rarely reached a body size of two meters in maximum length (Kriwet & Klug, 2004, 2015; see also Pimiento et al., 2019), some hybodontiforms easily exceeded their phylogenetically more derived relatives reaching an estimated maximum body size length of up to three meters, in particular those that are known to have predominantly inhabited open marine environments, such as *Asteracanthus*, *Planohybodus* and *Strophodus* (e.g., Underwood, 2002; Leuzinger et al., 2015, 2017; Citton et al., 2019; Szabó & Főzy, 2020; Stumpf et al., 2021). By contrast, the rather limited facies distribution of small-bodied hybodontiforms suggests that they predominantly inhabited marginal marine environments with reduced or fluctuating salinities (e.g., Duffin & Thies, 1997; Kriwet, 2004; Vullo et al., 2014).

Unlike most similarly sized hybodontiforms, *Durnonovariaodus maiseyi* gen. et sp. nov. appears to have predominantly inhabited deeper-water environments, possibly along the outer continental shelves and upper continental slopes, as also suggested for the rare, dentally similar species *Secarodus polyprion* from the Bathonian of England (Rees & Underwood, 2008). Both species may have occasionally moved to more shallow water environments for feeding, similar to modern hexanchiform sharks, which are generally bound to deep-water environments, but sometimes also occur in inshore continental waters (Ebert, Fowler & Compagno, 2013; see also Priede & Froese, 2013).

Hybodontiforms reported from the Late Jurassic Kimmeridge Clay Formation, aside from the holotype of *Durnonovariaodus maiseyi* gen. et sp. nov., are represented by teeth, isolated cephalic and dorsal fin spines, as well as partial skeletons attributable to different, predominantly large-bodied taxa (Woodward, 1889; Underwood, 2002, 2020). The precise systematic and taxonomic classification of these hybodontiforms, however, still remain problematic and unresolved in many cases, particularly given recently published efforts to better understand the diversity of Mesozoic hybodontiforms (e.g., Rees et al., 2013; Leuzinger et al., 2017; Szabó & Főzy, 2020; Stumpf et al., 2021), pending further research. According to current available data (Underwood, 2002, 2020; Stumpf et al., 2021; this study), hybodontiforms from the Kimmeridge Clay Formation include five large-bodied genera, comprising *Durnonovariaodus* gen. nov., *Planohybodus*, *Asteracanthus*, *Strophodus*, and *Meristodonoides*. The latter is represented by new, as yet unnamed species (Underwood, 2020) that extends the stratigraphic range of *Meristodonoides*, which was initially recognized in the Cretaceous (Underwood & Cumbaa, 2010), back to the Late Jurassic (see also Leuzinger et al., 2017). In addition, there is also a small-bodied hybodontiform, which is assigned by Underwood (2020) to *Hybodus lusitanicus* Kriwet, 2004, a species otherwise considered consistent with referral to the genus *Parvodus* Rees & Underwood, 2002 (Rees et al., 2013).

The genus *Planohybodus*, which formed a common and widely distributed constituent of Mesozoic marine ecosystems, apparently ranging from the Middle Jurassic to the Late Cretaceous (e.g., Rees & Underwood, 2008; Bermúdez-Rochas, 2009; Bourdon et al., 2011; Duffin & Sweetman, 2011; Alvarado-Ortega et al., 2014), was certainly among the most common hybodontiforms encountered in the Kimmeridge Clay Formation, as inferred from abundant but in many cases poorly preserved tooth crowns, which commonly co-occur with crowns of *Meristodonoides* (Underwood, 2020; SS pers. obs.). Although incomplete, these teeth can readily be distinguished from those of *Durnonovariaodus* gen. nov. by differences in tooth cusp morphology and ornamentation (Underwood & Cumbaa, 2010). Although tooth morphologies alone do not necessarily mirror feeding behaviours in chondrichthyans, those displayed by *Planohybodus* and *Meristodonoides* suggests that both taxa were adapted towards clutching and tearing rather than cutting prey. The disjunct monognathic heterodonty displayed by *Durnonovariaodus* gen. nov. suggests that the symmetrical, more gracile anterior teeth were probably used for capturing and handling prey, while the asymmetrical teeth from lateral and posterior positions more likely performed a cutting function. Therefore, based on the current available data, it seems likely that within the European Late Jurassic marine communities *Durnonovariaodus* gen. nov. has occupied an ecological niche distinct from that of other morphologically and presumably ecologically similar hybodontiforms, probably in order to avoid risk of direct competition.

## Conclusions

Hybodontiforms have an extensive fossil record elucidating a speciose clade of Palaeozoic to Mesozoic shark-like chondrichthyans that have developed diverse dental adaptations in relation to prey and feeding. However, even after almost two centuries of research, the taxonomy and systematics of hybodontiforms still remain poorly understood. This is mainly due to the scarcity of well-preserved skeletal material, which commonly provide important morphological features for inferring phylogenetic interrelationships.

The Etches Collection, which is now housed and curated in the Museum of Jurassic Marine Life of Kimmeridge, England, contains well-preserved but largely unstudied hybodontiform skeletal material from the Upper Jurassic Kimmeridge Clay Formation of southern England, including a partial skeleton of a comparably large-bodied hybodontiform, which is here described and designated as a new genus and species, *Durnonovariaodus maiseyi* gen. et sp. nov., and which significantly adds to our limited understanding of the diversity, ecology and distribution of Late Jurassic hybodontiforms.

The holotype and only known specimen of *Durnonovariaodus maiseyi* gen. et sp. nov., which could not be assigned to a particular gender given its incomplete preservation, shows a puzzling combination of dental and skeletal characters. Although skeletally similar to the better-known genera *Hybodus* and *Egertonodus*, which are traditionally referred to the family Hybodontidae due to close dental and skeletal similarities, teeth of *Durnonovariaodus* gen. nov. exhibit a unique combination of morphological characters reminiscent of *Secarodus*, which was erected to include distinctive, strongly labio-lingually compressed multicuspid cutting teeth from the Bathonian of England originally described as *Hybodus polyprion*. Skeletally, *Durnonovariaodus* gen. nov. resembles *Hybodus* and *Egertonodus* in possessing a palatoquadrate with a palatobasal process and an ethmoidal articular surface and in having costate dorsal fin spines. These features, however, contrast with *Asteracanthus*, whose teeth are otherwise rather more similar to those of *Hybodus* and *Egertonodus* than to those of *Durnonovariaodus* gen. nov. and *Secarodus*, rendering the perception of currently available phylogenetic hypotheses of hybodontiforms difficult and unresolved, which consequently led us to tentatively refer *Durnonovariaodus* gen. nov. to Hybodontidae.

The holotype of *Durnonovariaodus maiseyi* gen. et sp. nov. preserves two unfused pelvic half-girdles, a feature that has previously been considered as evolutionary primitive among hybodontiforms. However, unlike previously described, separate pelvic half-girdles also occur in the supposedly closely related species *Hybodus hauffianus* and may in fact have been more widely distributed among hybodontiforms than previously thought, thus rendering the phylogenetic utility of unfused pelvic half-girdles for inferring hybodontiform interrelationships difficult and unresolved.

All these discrepancies can only be countered by conducting more comprehensive comparative studies focusing on hybodontiform species that are presented by dental and skeletal material, combined with a subsequent phylogenetic analysis utilizing robust cladistic principles. These future studies will focus not only on new, yet largely unstudied hybodontiform skeletons from the Late Jurassic Kimmeridge Clay Formation, but also on historically collected skeletons from the Early Jurassic Posidonienschiefer Formation of Germany such as those referred to *Hybodus hauffianus*, which are in urgent need of re-investigation.

## Institutional abbreviations

MJML: Museum of Jurassic Marine Life, Kimmeridge, Dorset, England
NHMUK: Natural History Museum, London, England
SMNS: Staatliches Museum für Naturkunde Stuttgart, Germany

## Additional Information and Declarations

### Data Availability

The following information was supplied regarding data availability: Specimen shown in Figs. 2–6 is housed in the Museum of Jurassic Marine Life, England, and catalogued under MJML K1624. Specimens shown in Figs. 7A–7D are stored in the Staatliches Museum für Naturkunde Stuttgart, Germany, and catalogued under SMNS 10060 and SMNS 15150, respectively. Specimen shown in Fig. 7E is housed in the Natural History Museum, London, England, and catalogued under NHMUK PV P 339.

### New Species Registration

The following information was supplied regarding the registration of a newly described species: Publication LSID: urn:lsid:zoobank.org:pub:21199B53-9E93-44FF-987C-EDC115E8AA88. Durnonovariaodus gen. nov. LSID: urn:lsid:zoobank.org:act:35DA49B9-B14E-4390-BEA3-9B99C9D9BAB0. Durnonovariaodus maiseyi gen. et sp. nov. LSID: urn:lsid:zoobank.org:act:2E9EAEC3-16FA-4304-A6FE-1123135A46BF.
